# Solving the Orientation Specific Constraints in Transcranial Magnetic Stimulation by Rotating Fields

**DOI:** 10.1371/journal.pone.0086794

**Published:** 2014-02-05

**Authors:** Assaf Rotem, Andreas Neef, Nicole E. Neef, Andres Agudelo-Toro, David Rakhmilevitch, Walter Paulus, Elisha Moses

**Affiliations:** 1 Department of Physics and SEAS, Harvard University, Cambridge, Massachusetts, United States of America; 2 Bernstein Center for Computational Neuroscience, Goettingen, Germany; 3 Department of Clinical Neurophysiology, University Medicine Goettingen, Goettingen, Germany; 4 Department of Neuropsychology, Max Planck Institute for Human Cognitive and Brain Sciences, Leipzig, Germany; 5 Max Planck Institute for Dynamics and Self-Organization, Goettingen, Germany; 6 Chemical Physics Department, Weizmann Institute of Science, Rehovot, Israel; 7 Department of Clinical Neurophysiology, University Medicine Goettingen, Goettingen, Germany; 8 Department of Physics of Complex Systems, Weizmann Institute of Science, Rehovot, Israel; Cardiff University, United Kingdom

## Abstract

Transcranial Magnetic Stimulation (TMS) is a promising technology for both neurology and psychiatry. Positive treatment outcome has been reported, for instance in double blind, multi-center studies on depression. Nonetheless, the application of TMS towards studying and treating brain disorders is still limited by inter-subject variability and lack of model systems accessible to TMS. The latter are required to obtain a deeper understanding of the biophysical foundations of TMS so that the stimulus protocol can be optimized for maximal brain response, while inter-subject variability hinders precise and reliable delivery of stimuli across subjects. Recent studies showed that both of these limitations are in part due to the angular sensitivity of TMS. Thus, a technique that would eradicate the need for precise angular orientation of the coil would improve both the inter-subject reliability of TMS and its effectiveness in model systems. We show here how rotation of the stimulating field relieves the angular sensitivity of TMS and provides improvements in both issues. Field rotation is attained by superposing the fields of two coils positioned orthogonal to each other and operated with a relative phase shift in time. Rotating field TMS (rfTMS) efficiently stimulates both cultured hippocampal networks and rat motor cortex, two neuronal systems that are notoriously difficult to excite magnetically. This opens the possibility of pharmacological and invasive TMS experiments in these model systems. Application of rfTMS to human subjects overcomes the orientation dependence of standard TMS. Thus, rfTMS yields optimal targeting of brain regions where correct orientation cannot be determined (e.g., via motor feedback) and will enable stimulation in brain regions where a preferred axonal orientation does not exist.

## Introduction

More potent therapies are needed for mental illnesses such as mood disorders, schizophrenia and anxiety disorders which are common, restricting and expensive. One potential candidate to improve therapies is Transcranial Magnetic Stimulation (TMS), a noninvasive brain stimulation that uses a brief magnetic pulse to induce a transient electric field in the underlying neural tissue. Repetitive TMS modulates the responsiveness of cortical neurons and coupled circuits, which can result in an improvement of mental disorders [1]–[5]. Despite the extensive body of literature on the therapeutic effects of TMS on psychiatric diseases, the impact or persistence of these effects remain controversial and the only treatment application of TMS approved by US Food and Drug Administration is to depression [6]. Therefore, to maximize the therapeutic value of this noninvasive method improvements are needed [7]. We suggest viewing this task as challenges in two separate areas: one is the advancement of rational design of stimulus protocols; another is the successful implementation of stimulation in a given human subject.

**Figure 1:**
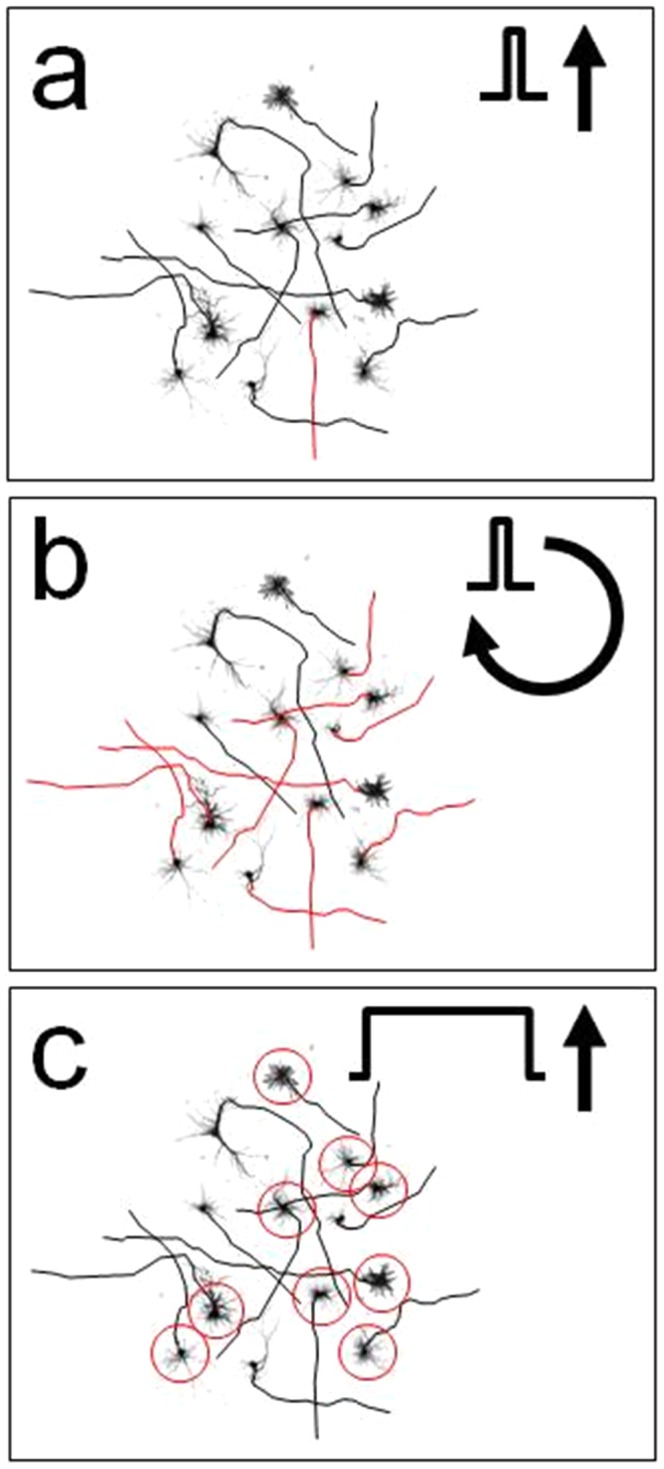
Schematic of a culture whose cell's axons (red or black lines) are randomly orientated. a) A short magnetic pulse with a fixed single orientation (black arrow indicates direction of the stimulating field) stimulates only one cell whose axon (red line) is oriented parallel to the direction of the induced electric field is excited. b) A short rotating magnetic pulse (arc indicates the span of rotation of the stimulating field) stimulates all cells whose axons' orientations lie within the arc of the rotating electric field (red lines), leading to a population response of the network. c) Alternatively, when applying a long magnetic pulse with a fixed orientation, all cells with dendrites oriented parallel to the direction of the induced electric field (red circles) are excited, leading to a population response of the network. See also Note S1, S2 and S3 in File S1.

In search of more effective and longer lasting TMS-effects, stimulation parameters such as the pulse shape [8]–[10], intensity and repetition rate have been widely varied in experiments on human subjects. To advance directed, rational design, a better understanding of the biophysics of magnetic stimulation and the physiology that underlies ensuing excitability changes is needed. While human experiments are typically limited to behavioral observations, in-vitro and in-vivo models allow the use of invasive and pharmacological techniques and will therefore significantly help to unveil the biological mechanisms of brain response to TMS. Previous studies report MEP-recordings from rodent limb muscles [11]–[15] and immunohistochemical evidence of the modulatory effect of rTMS on protein expression in rat cortical neurons [16]. However, although TMS in anaesthetized and awake rats is feasible, rat cortex is difficult to target with existing TMS systems [13]. Likewise, successful magnetic stimulation of neuronal cell cultures had not been reported until recently. It required elaborate treatment of the substrate to pattern cell growth such that axons are aligned with the induced electric field [17]. Even then, only a small subpopulation (about *1%* of the neurons in the culture) fired in direct response to the magnetic pulse, which limits the use of cell cultures further. To elicit a response in widely used non-patterned cultures would require a more global stimulation scheme that can target axons with different orientations (Figure 1). In conclusion, a technological development rendering standard cell cultures and rodents available for basic TMS research would remove a significant impediment in the field.

The second key step in improving TMS in human is the successful implementation of a given TMS protocol in the human subject. To achieve the best possible effect of stimulation it is not only necessary to choose a certain pulse shape and repetition rate [8]–[10], but also to adjust stimulation intensity, coil position and coil orientation in the plane tangentially to the head. Coil orientation has a major impact on the effect of stimulation because the magnitude and the polarity of the achieved neural modulation depend on tissue morphology, such as position and shape of tissue boundaries, and on cellular properties, such as axon diameter, myelination, orientation and curvature. Although modeling studies have started to address the interaction of subject-specific tissue morphology and coil position [18]–[21], to date, the subject's brain-morphology cannot be accounted for in a practical way and more pragmatic strategies are used to determine coil position. If stimulation of the target area elicits a direct response, a trial-and-error search can identify the optimal stimulus position and orientation. But, in therapeutic applications this approach is not available because cortical excitability is modulated through repetitive stimulation over the course of several minutes without any immediate feedback on stimulation success. The correct position of the coil can still be approximately derived with simple placement rules, such as following the EEG 10–20 system, or by neuro-navigation of the TMS coil registered to an MRI of the subject. However, the optimal *orientation* of the coil cannot be inferred in this way. Therefore, the coil orientation is usually held fixed to reduce the large parameter space of possible orientations. Given the strong influence that stimulus direction has a on the latency and amplitude of the evoked response [8], [22]–[24], the effect of a standard TMS stimulus can change from supra-threshold to sub-threshold for orientation changes as small as 45 degree. Clearly then, the use of a fixed orientation across subjects will lead to varying results. Thus, a technological development that reduces the sensitivity of stimulation to coil orientation would greatly improve the implementation of TMS protocols, rendering them more reliable and consistent.

Here we introduce a novel stimulation concept, rotating field (rf) TMS, that allows for a fundamental change in the spatiotemporal pattern of the induced electric fields. The electric field rotates, because it is created by superposition of two precisely timed biphasic pulses using coils that are oriented orthogonally to each other. We demonstrate that using rfTMS, neurons in the human primary motor cortex get activated for any orientation of the coil, while standard coils require precise orientation to induce supra-threshold stimulation. Similarly, neurons in the rat's motor cortex and neurons in primary cultures are excited with much higher certainty when using rotating field stimulation, making these model systems more widely available for studies on the underpinnings of TMS effects. For studies in rat and in cell cultures a cross coil configuration is used, while in human experiments a cloverleaf coil [25], [26] with modified current control is used to achieve the field rotation. Rotating Field TMS can provide a simple and universally applicable solution to two main challenges in TMS, sensitivity to orientation and availability of in-vivo and in-vitro models.

## Materials and Methods

### Ethics statement

rfTMS in human subjects was self-experimentation of W.P. and A.N. Participants provided their written consent to participate in this study. The ethics committee of the university clinics Goettingen agreed to self-experimentation of these two authors, as no dependency of any kind (financial, hierarchical etc.) exists with respect to other authors on the study. All animal procedures were approved by the Weizmann Ethics Committee (IACUC).

### Preparation of magnetic coils

The magnetic coils were manufactured both in our lab and by Magstim (Spring Gardens, UK). Our procedure used a polyester coated rectangular copper wire *0.254 mm* thick and *6.35 mm* wide (MWS Wire Industries, USA). Wires were turned on custom made frames, insulated with glass fibers and cast in epoxy made from *1* part Versamid *140* (Cognis) in *2* parts EPON *815* (Shell).

### Cross coil configuration

**Figure 2:**
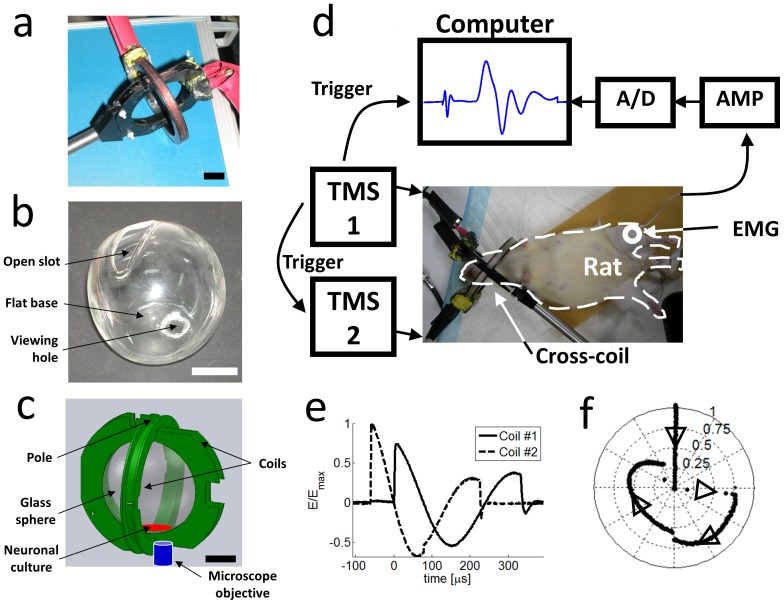
Cross coil experiments. a) A photograph of the cross coil used in the experiment. The two coils interlock on perpendicular planes and connect to two independent stimulators. b) A photograph of the glass sphere that was custom made to fit inside the cross coil. The glass coverslip, on which the neuronal culture grows, and the fluid medium were inserted through a slot located at the top of the sphere. The coverslip lay on a flattened base at the bottom of the sphere and was observed via a viewing aperture, which was sealed with optically transparent glass. See also Video S1. c) Schematic of the setup – the coverslip (red) was placed in a glass sphere inside the cross coil while an inverted epi-fluorescence microscope monitored neuronal activity. Scale bars in a–c are *2 cm*. d) Cross coil setup for rat experiments. The rat's head was positioned inside the cross coil (in place of the glass sphere, which was not used). EMG electrodes recorded muscle potentials from the Gastrocnemius. The EMG data was digitized and synchronized with the rfTMS pulses to assess the motor response to rfTMS. e) The induced electric field in the cross coil was measured using a pick-up coil oriented first on the plane of one of the coils (solid line) and then on the plane of the second coil (dashed line). The Magstim stimulator was loaded to *100%* and the HMS was loaded with *3.5 kV* (see details in the Methods section). f) A reconstruction of the effective electric field created from the sum of the two perpendicular components measured in e) with the field of coil #1 directed along the x-axis and the field of coil #2 along the y-axis. The effective field was reconstructed for a specific location just inside the poles of the cross coil (‘Neuronal culture’ arrow in Figure 2d). The effective field completes *¾* of a spiral cycle during the magnetic pulses cycle, as indicated by the black arrows.

For the cross coil (see Figure 2) we used two circular coils with *10* and *11* turns and inner diameters of *75* and *62 mm* respectively. Each of the two coils was connected to an independent power source. The coils were positioned one inside the other, while keeping their planes perpendicular. The hotspot of the cross coil is located near the poles of the spherical construct, where the two coils intersect (Figure 2) and the induced fields of the coils are perpendicular to each other. Since the planes of the two coils are perpendicular, the cross coil does not suffer from mutual induction losses and it is simpler to calculate the electric fields it induces than in the cloverleaf coil.

### Cloverleaf coil configuration

**Figure 3:**
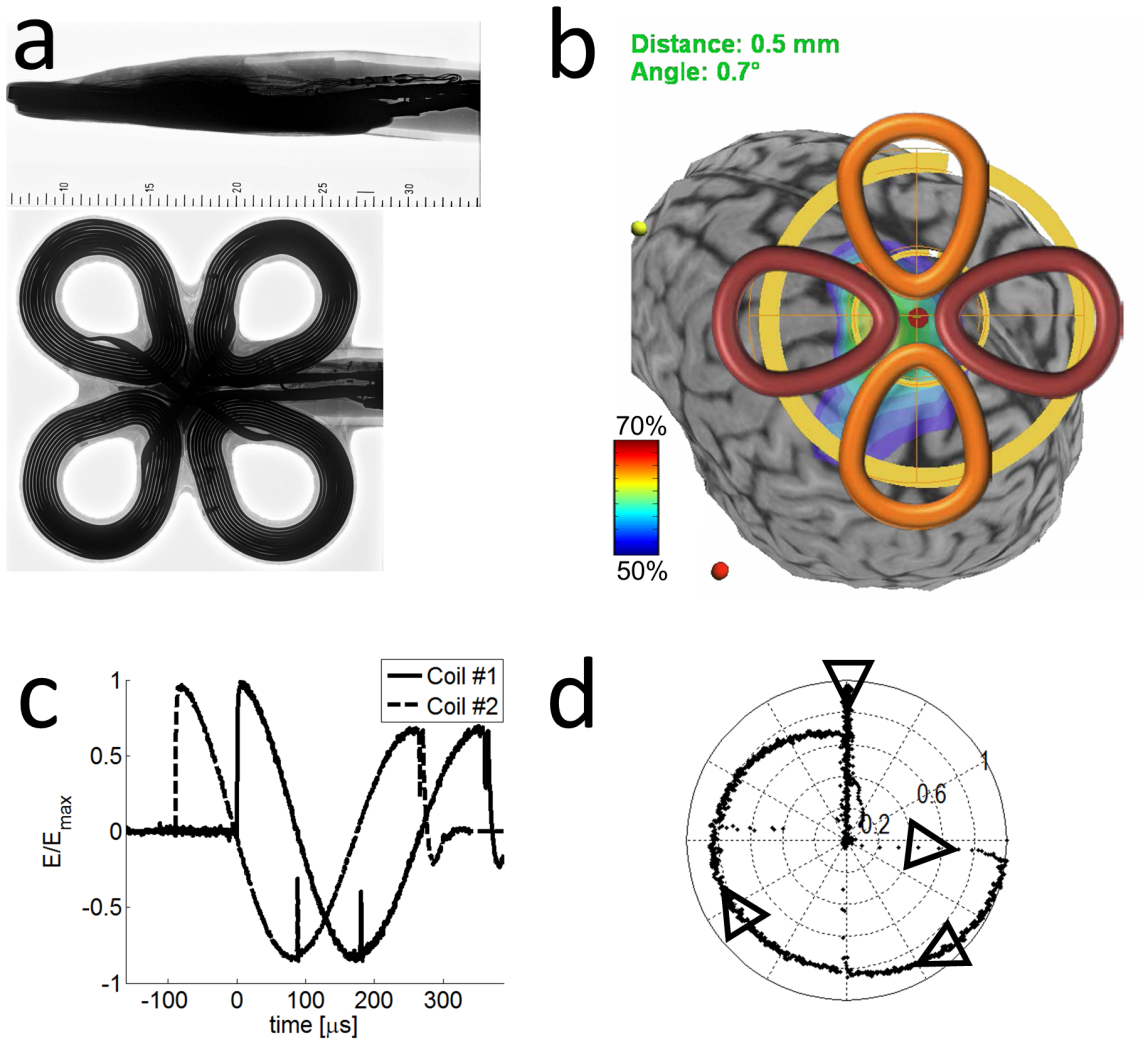
Cloverleaf coil experiment. a) Bottom and side X-ray images of the cloverleaf coil used in the experiment. The coils are coupled diagonally to form two figure of eight coils and each figure eight coil is connected to an independent power source. b) Neuro navigation software display. The position of the coil is tracked using the navigator and indicated by the central red dot and the yellow circle over an MRI scan of the brain of the subject. The coil shape is added offline to illustrate the actual position. The yellow sphere at the front is the nasion, the red sphere at the bottom left the left tragus, used in registering MRI and head position. The color scale indicates tentative field strength, calculated in real time assuming a spherical head model and a figure of eight coil. c) Electric field induced in a pickup coil positioned on 2 neighboring wings of the clover leaf coil. The coils were driven separately by 2 Magstim rapid^2^ stimulators. d) A reconstruction of the effective electric field amplitude and direction during a rfTMS pulse of the clover leaf coil with the field of coil #1 directed along the x-axis and the field of coil #2 along the y-axis.

The cloverleaf consists of two “figure of eight” coils, each connected to an independent power source. The coils are positioned on the same plane and are perpendicular to each other, so that at the hotspot their resulting electric fields are perpendicular. The shape of the coils is pointed, rather than circular, to produce high field strength in the center. Using simulations, we predicted the field shape for different coil shapes. The prototype cloverleaf manufactured by Magstim (Spring Gardens, UK) has a shape that provides an optimal compromise between field strength and homogeneity in the central region (Figure 3a).

### Power supplies

We used two independent power supplies in each experiment: for stimulating neuronal cultures and rat motor cortex we used a Magstim Rapid TMS (Magstim, UK) and a homemade stimulator (HMS) designed and manufactured in our lab. The HMS is based on a large *0.1* ****** *mF* capacitor (Maxwell Laboratories, USA) with a maximum voltage load of *22 kV*, and can produce magnetic (and induced electric) fields that are five times stronger than the fields delivered by the commercially available Magstim Rapid. To achieve accurate phase lag between the two magnetic pulses, the two power supplies were synchronized using a function generator (3390, Keithley instruments, USA). The signal generator issued two trigger signals separated by *1/4* of a cycle. This lag changed according to the coils used and ranged between *50–150 µs*. For the pilot experiments on humans we used two commercial Magstim Rapid power supplies, and a custom-made delay line was used to adjust the lag to 92 µs.

### Measurement of induced electric field and calibration of the coils

We used a small pick-up coil *40 mm* in diameter to measure the induced electric field of the coils. The pick-up coil was positioned inside the measured coil parallel to its plane, and was used to calibrate the cross coil as described in Note S4 in File S1.

### Calculations of the induced electric field

The induced electric fields for the cross coil were simulated using COMSOL (COMSOL Multiphysics 3.5, www.comsol.com, 2005). We used the Eddy Currents 3D model with the geometry and pulse profiles taken from the actual experiment (File S1). The electric field produced by the cloverleaf coil was qualitatively calculated using the magnetic vector potential as described in Note S5 in File S1.

### Preparation of primary cultures

Cultures were prepared from dissociated hippocampus of prenatal rats following a previously reported protocol [27]. Cells were plated on *30 mm* #0 glass coverslips (Menzel-Glaser, Germany), at a density of *3* million cells per coverslip.

### rfTMS of primary cultures

Cultures were stained with a calcium sensitive fluorescent dye (Fluo4, Invitrogen) and the calcium transients [17] imaged during application of magnetic pulses. The culture was placed in recording solution [17] that filled a near-spherical glass ball (Figure 2b), approximately *60 mm* in diameter, whose bottom was flattened to create a circular base approximately *30 mm* in diameter on which the coverslip lay. At the top of the sphere a slot was opened through which the coverslip and fluid could be inserted and at the base of the sphere a viewing hole *13 mm* in diameter was made, slightly off-center and near the circumference of the base, which was then sealed with an optically transparent glass coverslip. The glass sphere was placed inside the cross coil, with the flattened base positioned over the lower pole (Figure 2c and Video S1). The magnet and sphere were placed in an inverted microscope (Zeiss Axiovert 135TV), with the objective positioned under the viewing hole.

### rfTMS of anesthetized rats

During the TMS protocols, rats were anesthetized (see Note S6 in File S1) and positioned so that their motor cortex was at the focus of stimulation: the head was placed inside the cross coil, with the motor cortex located just below one of the poles. To monitor the effect of TMS on the rat, we recorded evoked muscle potentials from its hind legs using an Electromyogram (EMG) system (ActiveTwo, Biosemi, Netherland). We measured the stimulation threshold, defined as the minimal magnetic field required to create a response of more than *10 mV* as recorded in the EMG.

### rfTMS of human motor cortex

The subject relaxed in a reclining seat. A 3D localizer (ANT, Netherlands) was strapped to the head. Position and orientation of the coil was monitored using the neuro-navigation software *Visor* (ANT). The MRI image of the subject's brain was initially registered by reference points (nasion, left and right tragus) and skull tracing. For short experiments (2 orientations), the coil was fixed by a holder. For longer experiments (5 orientations 2 coils) the coil was positioned relative to the subjects head by a robot (ANT). In all settings the neuro-navigation software *Visor* was used to monitor the stability of the positioning. In all cases, the software reported that target position was met to within 1 mm and 3°. TMS, recording and analysis of muscle evoked potentials followed standard procedures. Surface EMG was recorded with Ag/AgCl cup electrodes in a belly-tendon montage from the right first dorsal interosseous muscle (FDI), band-pass filtered (0.002–2 kHz), amplified (Digitimer D360, UK) and sampled at 5 kHz (CED Micro 1401 mk II, Cambridge Electronic Design, UK). EMGs were analyzed in Igor-Pro (Wavemetrics, USA). The rfTMS coil was connected to two Magstim rapid^2^ stimulators (Magstim). For biphasic TMS a standard 70 mm figure-8 coil was used (Magstim #9925). For monophasic stimulation it was connected to a Magstim200 stimulator (Magstim). The motor hot spot of the left primary motor cortex was identified as point for optimally eliciting motor evoked potentials (MEPs) in the FDI. Then stimulation intensity was adjusted to elicit MEP-peak-to-peak amplitudes of 1 mV and kept constant throughout the experiment.

## Results

### Rotating magnetic field measurements

We measured the electric field induced by the cross coil using a circular pick-up coil positioned in the plane of the cross coil. The measurement was carried out first for the assembled cross coil, with the probe rotated by *90°* between measurements, and then separately for each of the two coils that construct the cross coil. The resulting phase shifted fields are shown in Figure 2e. The total resulting electric field performs a rotation, of which *270°* are scanned smoothly over three quarters of a cycle, lasting on the order of *300 µs*. During the first quarter of the cycle, the maximum magnitude of the rotating field is kept within 15% of the peak field strength obtained with a single coil driven by the Magstim power supply, as depicted in Figure 2f. The equivalent calibration was also performed for the clover leaf coil (Figure 3c and d).

### Computer simulation of the induced electric field

**Figure 4:**
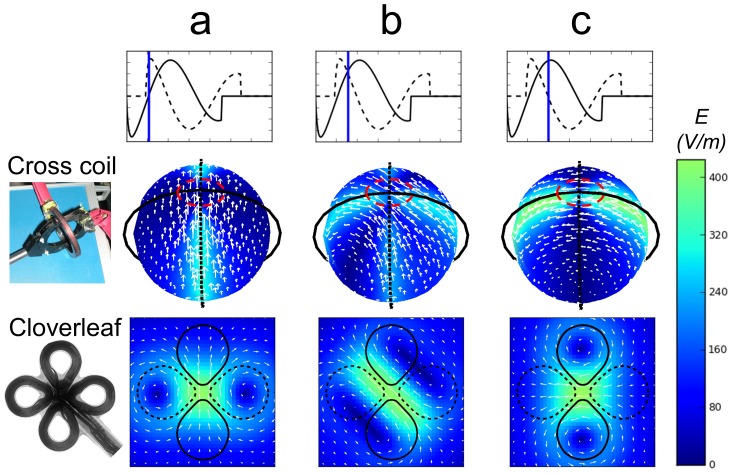
Simulations of rotating electric fields induced in the crossed coil and cloverleaf coil (photograph and X-ray image of the coils are presented at left of second and third row respectively). Upper row: idealized, calculated voltage traces – dashed line represents the voltage load on the dashed coils shown in the middle and bottom rows, solid line represents the voltage load on the solid coils. Blue vertical bars denote the time point for which the fields below were calculated. Middle row, cross coil: two circular coils are connected to two independent current sources each producing a single sinusoidal pulse (as described in the top row). The resulting electric field on the surface of a sphere positioned inside the coils is simulated (magnitude according to color code, direction by white arrows). a) After the solid coil completes *¼* of a cycle, the dashed coil commences its pulse and dominates the induction. b) A quarter of a cycle later, both coils induce an equal field and the effective field is diagonal. c) After another *¼* of a cycle, the solid coil completely takes over again and the resulting field is rotated by *90°* with respect to the original orientation in a). During a full cycle the orientation of the induced field on the sphere surface at the crossing point of the two coils (“hot spot”, red dashed ellipse) rotates, sweeping *270°*. Bottom row, cloverleaf coil: Two pairs of modified figure eight coils are connected to two independent current sources each producing a single sinusoidal pulse (the voltage load on the coils is described in the top row). The resulting electric field at a plane located *3 cm* above the coil and parallel to it is simulated (magnitude according to color code, direction by white arrows). a) After the solid pair completes *¼* of a cycle, the dashed pair commences its pulse and dominates the induction, resulting in a vertical field. b) *¼* of a cycle later, both coils induce an equal field and the effective field is diagonal. c) After another *¼* of a cycle, the solid pair completely takes over and the resulting field is horizontal. During a full cycle the orientation of the induced field at the center of the cloverleaf (“hot spot”) rotates, sweeping *270°*. See also Video S2.

We simulated the electric field induced by both the cross coil and the cloverleaf coil for different phases of the rotation cycle. The electric field induced in 3D by the cross coil is simulated over a sphere placed inside the coils and is maximal at the poles where the two coils intersect (Figure 4b). This is the hotspot for maximal excitation and rotation, and is where we located the culture and the motor region of the brain of the rat. The electric field induced by the cloverleaf coil is simulated in 2D over a plane located 3 cm below the coils and is maximal under the center of the cloverleaf. This is the hotspot for maximal excitation and rotation, which is where we located the motor region of the human subject's brain (figure 4c). We used these simulations to estimate the induced electric field at the hotspots, where the maximum rotating field that our system could induce was of the order of *300 V/m*. In real applications charge accumulation will decrease this value and change the primary E-field direction.

### Excitation of 2D neuronal cultures

The main result of using the cross coil is immediately seen by applying it on two dimensional (2D) cultures. While we were previously unable to excite 2D cultures with magnetic pulses, in the cross coil this was rather easily achieved. This is readily understood from simulations of neuronal excitation by conventional TMS and rfTMS, performed in NEURON (Note S7 and Figure S1 in File S1). As shown in Table 1, half of the 2D cultures tested (*15* out of *N = 30*) were excited by magnetic stimulation. The electric field threshold for excitation had a mean of *90±10%* (SD) of the maximum rotating field. According to our estimation the corresponding electric field induced at the hotspot is 270 *V/m* which agrees with that reported previously for 1D cultures [17] (*300±130 V/m* (SD)). Surprisingly, with this geometry *13%* (*N = 4*) of the 2D cultures also responded to single coil excitation, with a threshold field that was between *20%* and *50%* higher than the threshold for cross coil stimulation (we used the HMS to induce non-rotating electric fields that were up to 2 times stronger than the maximum rotating field).

**Table 1:** Summary of magnetic stimulation response in both neuronal 2D cultures and anesthetized rats.

	2D culture (N = 30)		Rat (N = 9)	
	Responded to stimulation	Electric Threshold (Normalized to maximum inducible field)	Responded to stimulation	Electric Threshold (Normalized to maximum inducible field)
Cross coil	50%	90±10%	89%	70±10%
Single coil	13%	120*±20%	44%	90±10%

All distribution errors are standard error (SE). % denotes fraction of the maximum attained rotating electric field.

* When using only our home made stimulator as the single coil we can induce non-rotating electric fields that are up to 2 times stronger than the maximum rotating electric field.

A qualitative test for the directionality is found in two cultures that were excited both by a single coil and the cross coil. By physically rotating the culture *45°* with respect to the coil we could check whether the initial random orientation was dominant in enabling the excitation. The single coil stimulation was indeed sensitive to this rotation, with the threshold climbing beyond the maximum field strength of our system. Strikingly, stimulation with the cross coil showed no sensitivity to the rotation, and the culture responded at all angles (*0°*, *45°*, *90°*, *135°*, *180°*, *225°*, *270°* and *315°* with respect to one of the coils constructing the cross coil), qualitatively demonstrating the alleviation of the directional sensitivity by rfTMS.

### Excitation of rat motor cortex

**Figure 5:**
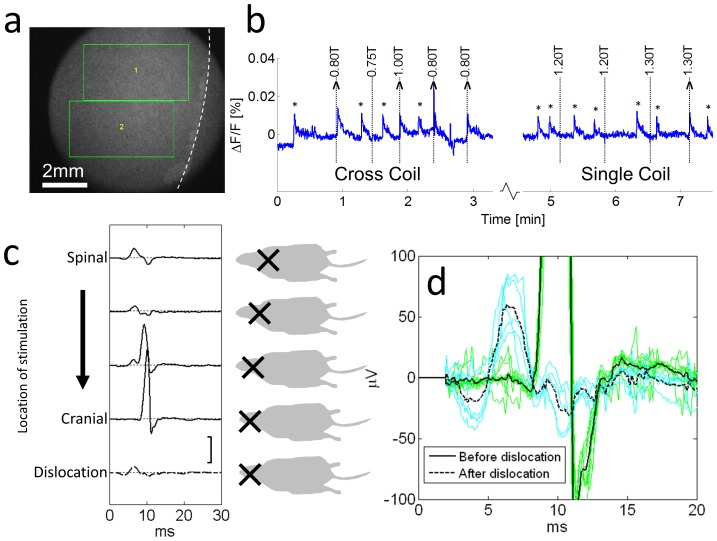
Results from neuronal culture and from rat motor cortex. a) The response of 2D Neuronal culture to rfTMS. Spiking activity in the culture was imaged through the viewing aperture (see Figure 2b). Fluorescent neurons are seen as white spots. The green rectangles indicate the regions of interest over which the signal was averaged. The dashed white line indicates the borders of the coverslip on which the culture was grown. b) The average calcium dependent fluorescence of the regions of interest outlined in a). Dashed lines mark events of magnetic stimulation using first the cross coil and then a single coil. The intensity of each stimulation pulse is noted in Tesla. Successful stimulation of a population response is indicated by a caret while intrinsic activity unrelated to magnetic stimulation is indicated by an asterisk. Note that the cross coil successfully triggered a response already with 0.8T (but not at 0.75T), while a single coil required around 1.3T. c) The response of rat motor cortex to rfTMS. Graphs of EMG recording of the Gastrocnemius when using the cross coil to stimulate a rat at different locations. Each location is illustrated to the right of the response trace with the black cross representing the cross coil. The last row was performed after cervical dislocation of the rat. Scale bar is *200 µV*. d) A comparison between the last two rows in c). The solid line is the average of *10* individual responses (green traces) of the rat to rfTMS over its head before dislocation and the dashed curve is the average of *5* individual responses (cyan traces) of the rat to rfTMS in a similar location over its head after dislocation.

The cross coil configuration is particularly well suited for application on rats, since the head of the animal fits nicely inside the cross coil, with the cortex located where the field is maximal, near the joint axis of the two coils (see Figure 5c). We tested *9* anesthetized animals for the response of the Gastrocnemius muscle to magnetic stimulation, as measured by an EMG electrode on the leg of the animal. The major difficulty in this test is to differentiate between the excitation of the motor cortex and that of the spinal cord. This was done using the different latencies of the response in the two excitation modes.

As shown in Figure 5d, the response of the Gastrocnemius to stimulation was complex yet reproducible. Two typical latency times were observed, and we associated the shorter one with the spinal response (*3.2±0.2 ms* (SE)) and the longer one with the cortical response (*7.4±0.4 ms* (SE)). In most cases, the spinal and cortical responses could thus be reliably differentiated by the latency time. Cervical dislocation or sectioning of the spine abolished the longer latency response, while leaving the shorter one active for several minutes. The spinal response was typically achieved at a lower magnetic stimulation threshold than the cortical one. We observed a clear cortical response in eight of the nine animals tested. The estimated electric field threshold for excitation was distributed around a mean of *70±10%* (SD) of the maximum inducible field.

Four of the animals also responded to stimulation using only a single coil of the cross coil pair. As in the neuronal culture stimulations, when using only a single coil the electric field threshold was between *10%* and *50%* higher than that of the cross coil system and exhibited a strong directional dependence as expected.

### Excitation of human motor cortex

**Figure 6:**
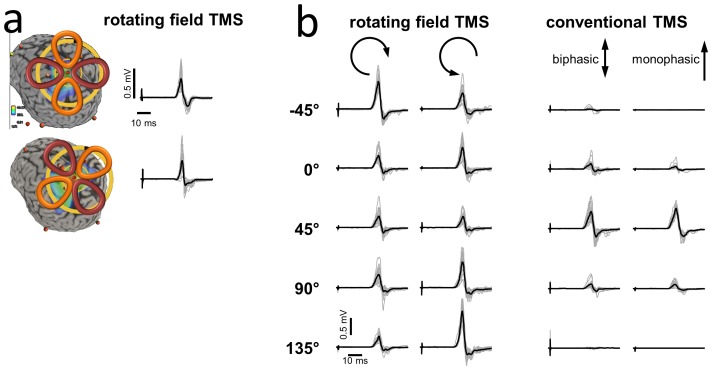
The response of Human motor cortex to rfTMS. a) The cloverleaf coil is positioned over the motor cortex. Stimuli with constant strength are applied at 0.2 Hz. Individual muscle evoked potentials recorded from the first dorsal interosseous are shown in grey, and the averaged response in black. Rotating the coil by 50° in the plane of the cloverleaf coil and maintaining the center position does not change the amplitude of the muscle response. Data obtained from subject A.N. b) Extended experiment, testing 5 different orientations, controlled by a robotic device. For each orientation two opposite rotation directions of the rotating field were tested. The experiment was then repeated using a standard figure-eight coil, used to apply both biphasic pulses, as in rfTMS, and mono-phasic pulses. Under standard TMS no muscle response can be detected for orientations more than 45° away from the optimum. In contrast, rfTMS provides reliable stimulation independent of the coil's orientation. Data obtained from subject W.P.

The clover leaf magnetic coil was used to demonstrate the efficacy of rfTMS on human subjects. The location and orientation of the coil and the corresponding motor response is depicted for two positions in Figure 6a. Clearly, the two orientations, rotated with respect to each other by an angle of 50°, elicit the same response. Using a robotic stereotactic device controlled by a neuro-navigation software, the experiment was repeated for 5 orientations, covering a rotation by 180°. The same location in the motor cortex was stimulated with the rfTMS clover leaf coil and with a standard figure-eight coil. While the motor response evoked by the standard TMS coil drops dramatically for orientations away from the optimal orientation, rfTMS can elicit a response at any orientation.

A slight orientation dependence remains in the motor response to the cloverleaf coil. This is expected because the induced electric field traces out only 270° of a full circle (see Figure 1e) and can be seen also in the accompanying simulations (Video S2). For the same reason, the clockwise and counterclockwise rotations show a slight asymmetry in motor response. The remaining directionality can be overcome in the future by appropriately designed power supplies, building on recent advances in this field [9].

## Discussion

Stimulation of neurons by magnetic pulses relies on current injection along neuronal processes and is therefore inherently direction-dependent. In this paper we have overcome this dependence via the application of a rotating field. We realized this using a superposition of two spatially orthogonal and temporally phase-shifted stimuli delivered by two custom made coil setups – one that that was specially designed for the stimulation of small-scale model systems and another for targeting the human cortex. In both cases, the rotating field had striking advantages.

Model systems are necessary to achieve a detailed understanding of the biophysical basis of TMS stimulation and the excitability changes it can induce. However, while thousands of studies of TMS application in humans are published, only a few dozen or so use the standard model systems of neuroscience: rodents and 2D cell cultures. We have shown that rotating field magnetic stimulation delivered by the cross coil reliably drives excitation in both model systems. The ability to stimulate 2D cultures and rat cortex magnetically is particularly significant in view of the reported difficulty to achieve this using the standard single or figure-eight planar coils [17] (see also Note S8 and S9 in File S1). Although the overall duration of the pulse is longer by 25% compared to non-rotating TMS, we believe that the improved stimulation is a direct result of the rotation of the field, and is consistent with our understanding that: 1) axons are the neuronal domain that is excited during TMS and 2) the axons have no preferred orientation in 2D cultures.

We were surprised to find that *4* out of *15* cultures that could be excited by the cross coil were also excited by only one of the coils that construct the cross coil (Table 1). This could occur if in those *4* cultures a sufficient number of axons were aligned by chance in the direction of the induced electric field (for an example see Figure S2 in File S1). Since the field of a one-coil magnet is spatially directed, it is not surprising that in those cases the excitation of the cultures was directional-dependent and could be abolished by rotating the culture by *45°* with respect to the coil. This demonstrates an obvious advantage of rfTMS. It eliminates the need for precise orientation of the coil, which is always time consuming if it can be achieved, but is often impossible to attain in humans because no direct readout of stimulus efficiency exists. Moreover, this “exception to the rule” demonstrates the rule - the probability that such an orientation exists, i.e. that several axons in the culture are oriented along a single axis, is presumably low. In all other cases the axon orientation is distributed randomly, and it is the scanning ability of the cross coil that enables the excitation of those cultures. Thus, rotating field does more than just find the right orientation and excite the axons that lie in that direction, it enables stimulation of neuronal structures whose axons are oriented in a random fashion, with no preferred directionality.

The comparison between rfTMS and the existing standard brain stimulation was performed by applying the cloverleaf coil to the index finger region of the primary motor cortex, showing that rfTMS induces clear response regardless of the coil orientation. This self-experimentation which is limited to the motor cortex somewhat underrepresents the power of rfTMS. Motor response is reliably induced with supra-threshold stimulation using conventional coils while in other regions such as the visual area, only a very narrow range of coil-orientations would have provided reliable, trial-by-trial supra-threshold stimulation. Moreover, without feedback such as muscle-evoked or vision-evoked potentials conventional TMS could not ascertain reliable stimulation, while rfTMS works almost regardless of coil orientation. Thus, rfTMS clearly holds great promise for an increased reliability in the magnetic stimulation of higher function cortex areas which do not provide immediate feedback. In addition, if there are regions of the brain where axon orientation is distributed randomly, standard TMS is not expected to elicit a response while the scanning ability of the cloverleaf coil should enable the excitation of such regions, as when applying the cross-coil to standard 2D cell cultures.

It should be emphasized that rfTMS as a technology is complementary in nature, and can be used in tandem with most other advances in TMS technology, e.g. deep TMS or novel repetitive frequency protocols. The additional power supply and the double magnets pose a minimal technical or financial burden, comparable to that incurred in existing paired-pulse setups, with advantages easily overcoming the cost.

Orientation free stimulus may not be adequate in all cases. Studies performed on the primary motor cortex indicate, that stimulus direction not only influences the threshold for stimulation, but can also select between different sub-populations of neurons that get excited [24], [28] and, more importantly, affect the ability to induce lasting modulation of cortical excitability [29]. The implications of these findings for the effect of rfTMS are not clear. On the one hand, coincident activation of several sub-populations might mask specific effects; on the other hand, long lasting modulations of equal sign, i.e. either potentiation or suppression, which are induced in each of several sub-populations individually could conceivably sum up to reach larger magnitudes than achieved with conventional, directional stimulation. In the clinical setting, selective activation of oriented subpopulations is not part of current practice, thus loss of directional specificity should not be a limitation. Instead, rfTMS would most probably warrant reliable stimulation, where standard TMS might fail to excite the target at all.

The sensitivity to field orientation has its origin in the directionality of axons, and in the fact that magnetic stimulation is achieved via axonal excitation. If the neuron could be excited at the dendrites then the dependence on field orientation would disappear (as in rfTMS) since the dendritic tree is isotropic (Figure 1c, see also Note S1 in File S1). Because of their different physical properties, excitation of dendrites necessitates the application of pulses with longer duration, but these are currently accessible only using electric excitation. We note that achieving long pulses in a magnetic stimulation is feasible, and is currently being pursued in our lab.

## Supporting Information

File S1 **Supplementary Notes and Figures.**
(DOCX)

File S2 **Comsol Model of the electric field induced by the cross coil using the geometry and pulse profile used in the 2D culture experiment.**
(MPH)

Video S1 **A rotating 3D schematic view of the cross coil, including its assembly and dis-assembly into two circular coils.**
(AVI)

Video S2 **Simulation of the electric field induced by the cloverleaf coil.** the arrows represent vectors of the induced electric field while color codes local magnitude of the electric field, in arbitrary units.
(MPG)
