# Supplementary material for: Solving the Orientation Specific Constraints in Transcranial Magnetic Stimulation by Rotating Fields
Source: PLoS One. 2014 Feb 5;9(2):e86794. doi: 10.1371/journal.pone.0086794 (PMC3914799; doi:10.1371/journal.pone.0086794)
Supplement: File S1 — Supplementary Notes and Figures. (DOCX) [file pone.0086794.s001.docx]

Solving the orientation specific constraints in transcranial magnetic stimulation by rotating fields

Supplementary Information File S1

Note S1 in File S1 - Directionality, rise time and co-operativity constraints in magnetic stimulation

Note S2 in File S1 - Alleviating directionality - Rotating electric fields

Note S3 in File S1 - Alleviating directionality - Dendritic excitation.

Note S4 in File S1 – Calibration of the coils

Note S5 in File S1 – Calculation of the induced electric field of the cloverleaf coil

Note S6 in File S1 – preparation of anesthetized rats

Note S7 in File S1 - Calculating the threshold for neuronal excitation

Note S8 in File S1 - Using the conventional circular coil in culture experiments.

Note S9 in File S1 - Using the conventional circular coil in rat experiments.

Figure S1 in File S1 – The calculated orientation dependence of magnetic stimulation.

Figure S2 in File S1 – A fluorescent image of a GFP expressing neuron grown in culture.

# Note S1 - Directionality, rise time and co-operativity constraints in magnetic stimulation

The neuro-physics of external field stimulation can be simplified using the passive cable equation[^1^](#_ENREF_1), [^2^](#_ENREF_2) which calculates the voltage that an external electric field induces on a cable’s membrane (in this case the cable is a neurite) whose capacitance and resistance is known. This equation emphasizes the role of two relevant parameters in the stimulation of neurites. First, the neurons that respond strongest to the induced electric field are those whose neurite – axon or dendrite – lies parallel to the electric field. This observation is clear from theoretical considerations and has been the basis for obtaining the first ever magnetic stimulation of cultured neurons from the central nervous system (CNS) using one-dimensional cultures. Second, neurites whose membrane rise time is much longer than the rise time of the external pulses will not respond to the stimulation. Since the rise time of commercial magnetic stimulators does not exceed *100 µs* and the membrane rise time of dendrites is of the order of *1 ms* - excitation of neurons is almost exclusively initiated at the axon, whose rise time is on the order of *100 µs*. A more detailed derivation can be found in a previous paper[^3^](#_ENREF_3). Additionally, the collective response of a network of neurons either in the live brain or in-vitro cannot initiate from the firing of a single cell. Rather, it usually requires a critical number of initiating neurons that fire almost simultaneously in order to trigger a population burst that can be observed either in-vivo or in large neuronal cultures in-vitro. Details can be found in a previous paper[^4^](#_ENREF_4).

The three factors mentioned above limit a successful magnetic stimulation of a network of neurons: they require that several neighboring cells in the network will have axons oriented parallel to the induced electric field. However, the direction of the magnetic field of a given standard coil is fixed, and so is the induced electric field. This means that in a network where neurons have randomly oriented axons the amount of neurons that can be excited is very small, not enough for a collective response of the network (Figure 1a). This is the main reason why it is so hard to excite two dimensional cultures in-vitro, or cortical regions whose axonal orientation is not homogenous.

# Note S2 - Alleviating directionality - Rotating electric fields

In this paper we overcame the problem caused by the anisotropy of axon orientation by a time dependent electric field, scanning through a whole range of angles. The resulting electric field rotates in space during the pulse cycle, leading to the excitation of additional cells whenever it scans through their orientation. Since the cycle lasts no more than few hundred microseconds, all these cells are stimulated closely enough in time, ensuring a collective response of the network (Figure 1b).

# Note S3 - Alleviating directionality - Dendritic excitation.

A different approach to the problem would be the direct excitation of dendrites in the culture by applying pulses with durations of the order of *1 ms*. As opposed to axons, which usually project a long stretch at a single direction from the soma before branching, dendritic trees tend to branch extensively from the soma in an isotropic pattern. The option of directly exciting dendrites is thus extremely valuable since it overcomes the mentioned problem of directionality – each cell has numerous dendrites oriented in almost all possible directions and will respond to an induced field in a fixed direction (Figure 1c). This solution may prove superior to the rotating field as it will enable targeting areas in the brain where all axons are oriented perpendicular to the cranial plane. Such neurons would not be excited even by rfTMS since the induced field in the cross and cloverleaf coils can only be made to rotate in a plane parallel to the skull.

# Note S4 – Calibration of the coils

To measure the induced electric field of the coils, we used a pick up coil 40 mm in diameter. The pick-up coil was positioned inside the measured coil, parallel to its plane. We used the pick-up coil to calibrate the cross coil as follows: first, we measured the relation between the power setting of the stimulator and the resulting induced field of each the coils and found a linear relation for each pair of stimulator and circular coil. Second, we compared the proportionality constant of the HMS coil vs. the Magstim coil and found that the induced field at 100% power setting of the Magstim was equivalent to 3 kV setting of the HMS. This determines the maximum intensity of the rotating field pulse along its first quarter, which for the cross coil was equal to 302±2 V/m at the hotspot of the coverslip plane (Figure 1e). In practice, since the pulse amplitude of both magnetic stimulators decayed by 15% for each ¼ of a cycle, we used the HMS device as the first stimulator and set its maximal power to 3.5 kV so that when the HMS device completes ¼ of its cycle the actual magnitude of the HMS component decays to a value equivalent to the maximum field component of the Magstim. We used this equivalence during our experiment, keeping the ratio of 3.5kV / 100% for any setting of pulse intensity. For example, when delivering a pulse that is half the maximum intensity, we used 50% Magstim power and 1.75 kV HMS load. When measuring fields induced by a single coil, we considered the average field during the first ¼ of the pulse cycle.

# Note S5 – Calculation of the induced electric field of the cloverleaf coil

The shape of the coil windings was discretized to straight segment vectors *l_i_* of 1 millimeter length. The height of the wire was accounted for by 6 parallel copies of the coil each conducting 1/6 of the total current. Each of the segments contributed to the magnetic vector potential A according to: $\mathbf{A}(\mathbf{r},t)=\frac{\mu_{0}}{4\pi}I(t)\sum_{segments\text{ i}} \frac{\mathbf{l}_{i}}{\left|\mathbf{r}_{i}-\mathbf{r}\right|}$, where *r_i_* is the vector pointing to the center of the segment *i*. The induced electric field is given by$\mathbf{E}(\mathbf{r},t)=-\frac{\partial\mathbf{A}(\mathbf{r},t)}{\partial t}$.

The precision of the numerical calculation was tested for idealized circular coils by comparison with the analytical solution. The discretization in segments of 1 millimeter length was sufficient to reproduce the analytical solution with less than 0.01% error in electric field intensity, assessed at a distance of 1 cm from the coil surface. Because of its symmetry, the cloverleaf coil does not suffer from mutual induction between the two figure of eight coils.

# Note S6 – preparation of anesthetized rats

We tested the effect of the new magnetic stimulators on adult rats anesthetized with Ketamine and Xylazine. Ketamine in combination with Xylazine has been shown to result in sufficient anesthesia and analgesia in rats without depressing vital functions[^5^](#_ENREF_5). All procedures were approved by the Weizmann Ethics Committee (IACUC). Prior to the experiments rats were anesthetized via IP injection of *75 mg/kg* Ketamine (Kepro, Holland) and *7.5 mg/kg* Xylazine (Kepro, Netherland). Since Xylazine affects the TMS threshold for the first hour after its application (data not shown), the rats were anaesthetized approximately *1 hr* prior to the beginning of the experiment. Ketamine was thereafter repeatedly injected IP throughout the experiment at an average rate of *75 mg/kg/hr* according to the animal’s level of anesthesia. At the end of the experiment, the rats were euthanized with Pentobarbitone Sodium (CTS, Israel).

# Note S7 - Calculating the threshold for neuronal excitation

To judge the stimulation efficiency of the clover-leaf coil we estimated the expected excitation threshold for various orientations between coil and neurite. As described before^1^, we used a depolarization of *30 mV* as criterion for successful excitation. Using the electric field ***E*** induced by the clover-leaf coil *3 cm* above its center, the effect on the membrane potential *V* of a passive cable (*1 µm* diameter, *1 mm* length) was calculated as described earlier[^3^](#_ENREF_3), using the cable equation with a source term accounting for the field ***E***:

$\lambda^{2}\frac{\partial^{2}V}{\partial l^{2}}-\tau\frac{\partial V}{\partial t}-V=\lambda^{2}\frac{\partial E_{l}}{\partial l}$.

The axial length constant *λ = 384 μm* and the time constant *τ = 300 μs* were chosen to approximate the conditions of an unmyelinated axon[^3^](#_ENREF_3), the *l*-axis runs parallel to the cable and *E_l_* is the projection of ***E*** onto this direction. The cable ends are assumed to be sealed, implying the boundary conditions:

$\frac{\partial V(0,t)}{\partial l}=\frac{\partial V(L,t)}{\partial l}=0$.

Using the axial resistance *r_i_* the source term is equivalent to a current injection $I_{i}=\frac{1}{r_{i}}\frac{\partial E_{l}}{\partial l}$ with opposite signs at the two ends. To perform the simulation, the IClamp method of the simulation environment NEURON[^6^](#_ENREF_6) was used. For each angle between *x*-axis and neurite the induced field was calculated for a certain maximal coil current and hence a certain maximal amplitude of the *x*- and *y*-components of ***E*** (*E_x_^max^* and *E_y_^max^*). Next, the projection *E_l_* was used to calculate the resulting time course of the membrane potential. The calculation was repeated varying *E_x_^max^* and *E_y_^max^* in a binary search, to find the value at which the membrane was depolarized by *30 mV*, our criterion for successful excitation. The systematic variation of the angle and coil current as well as the communication of the respective current injection *I_i_* to NEURON were automated using Python[^7^](#_ENREF_7).


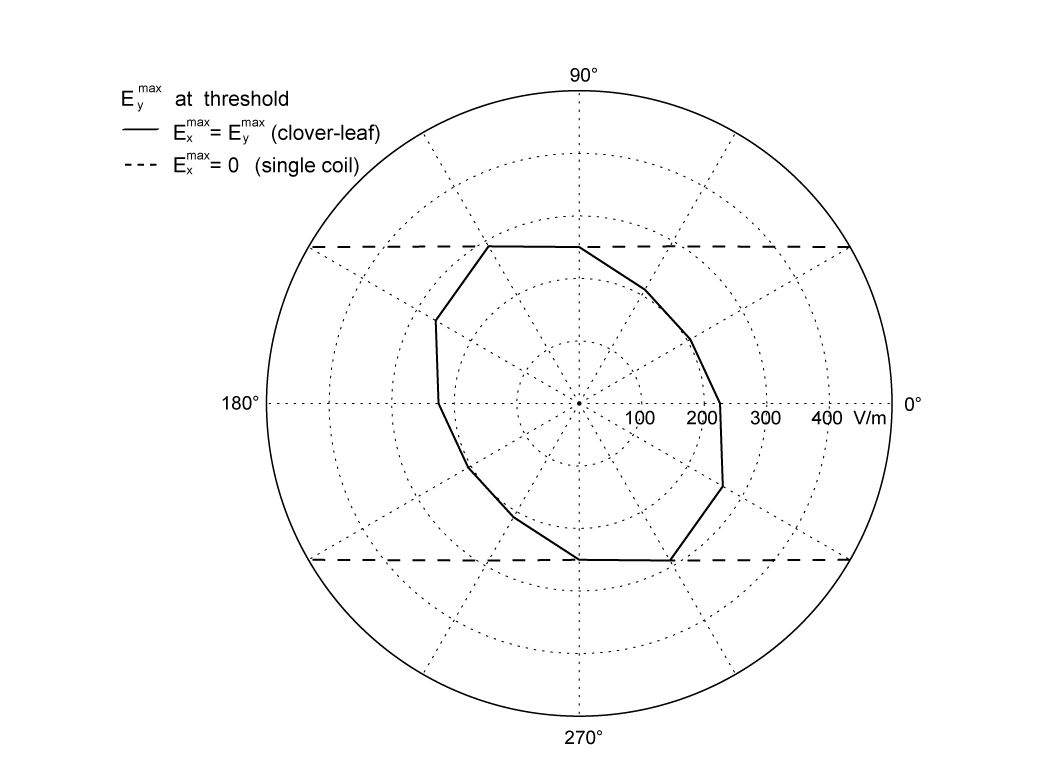


Figure S1 – The cloverleaf coil alleviates the orientation dependence of magnetic stimulation. The calculated electric field, induced by the dense cloverleaf coil (Figure 3, manuscript) was used to estimate the threshold for neuronal stimulation. The maximal amplitude *E_y_^max^* of the *E*-field’s *y*-component that was needed to reach threshold is plotted over the angle between *x*-axis and the neurite. With *E_x_^max^ = 0* the ‘standard’ configuration is implemented: only two opposed wings of the clover-leaf coil are used, forming a single figure of eight coil. This results in a threshold that is minimal when the neurite is aligned with the *y*-axis and steeply increases if the alignment deviates from this optimal orientation (horizontal dashed straight lines). In contrast, stimulation with the cloverleaf coil results in a threshold that changes by less than *50%* for all possible orientations of the axon.

# Note S8 - Using the conventional circular coil in culture experiments.

We previously reported that we were unable to magnetically stimulate randomly connected two-dimensional (2D) cultures using standard circular coils, even at high (*~5T*) magnetic fields produced by our homemade power supply[^3^](#_ENREF_3). This was based upon examination of *11* cultures. In the current study, we tested *16* of the *30* cultures with the standard horizontal circular coil and were surprised to find that *2* of the cultures responded not only to the cross coil but also to the standard coil (in addition to these *2* cultures, another *3* cultures were responsive to the standard coil but were not tested using the cross coil due to deterioration of the sample over time).

A possible explanation for this phenomenon is that in these cultures a subset of the axons is oriented in a direction parallel to the induced electric field. Since the electric field lies on rings concentric with the cover glass boundaries, we searched for axons that might lie in this direction. An example of one such neuron, marked with GFP, is presented in Supplementary Figure 1. The axon of this neuron extends to *3 mm* and grows parallel to the cover slip boundaries. The orienting effect of boundaries on axons has previously been demonstrated by us[^8^](#_ENREF_8). If indeed the boundary forces a large number of neurons to have axons oriented along the rim surrounding the culture then these would be excited by the induced electric field and could initiate activity in the whole culture.


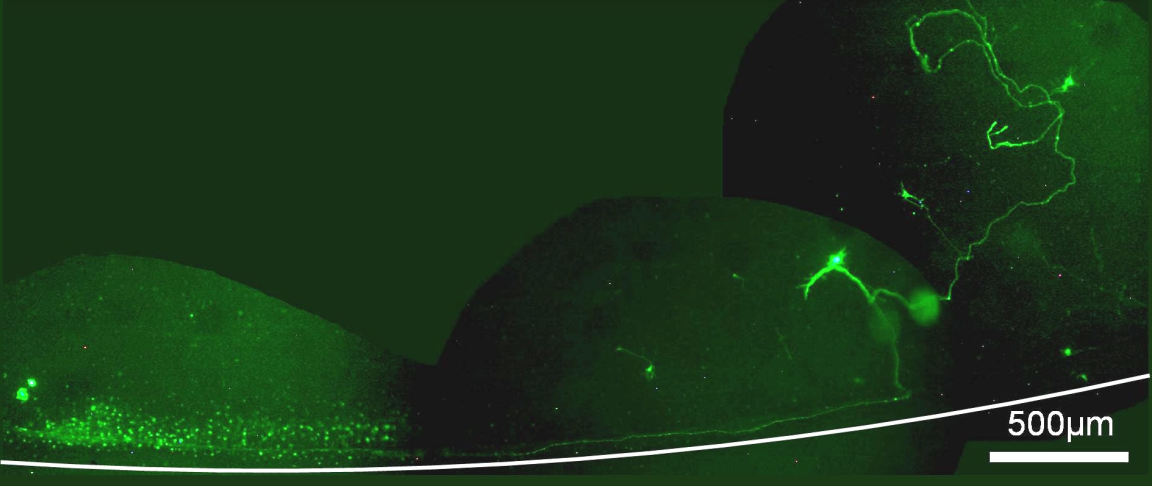


Figure S2 – A fluorescent image of a GFP expressing neuron grown in culture. The culture was plated on a *30 mm* coverslip (the white arc in the figure runs on the coverslip rim). The neuron’s axon can be tracked as it follows the coverslip circumference tangentially along *3 mm* (white arrow).

# Note S9 - Using the conventional circular coil in rat experiments.

In addition to the successful cross coil excitation of cortical activity in rats, as reported in the main text, we tried to magnetically stimulate rats using the standard single circular coil. While cervical excitation was evident, we were unable to differentiate a cortical component in the signal. We therefore mostly used the circular coil for the task of characterizing the effect of the anesthetic agents Ketamine and Xylazine on the threshold for excitation of the cervical activity. We also conducted one experiment using the cross coil to verify these results.

# References

1. Hodgkin, A.L. & Rushton, W.A. The electrical constants of a crustacean nerve fibre. *Proc Royal Soc. B* **133**, 444-479 (1946).

2. Roth, B.J. & Basser, P.J. A model of the stimulation of a nerve fiber by electromagnetic induction. *IEEE Trans Biomed Eng* **37**, 588-597 (1990).

3. Rotem, A. & Moses, E. Magnetic stimulation of one-dimensional neuronal cultures. *Biophys J* **94**, 5065-5078 (2008).

4. Breskin, I., Soriano, J., Moses, E. & Tlusty, T. Percolation in living neural networks. *Phys Rev Lett* **97**, 188102 (2006).

5. Zandieh, S., Hopf, R., Redl, H. & Schlag, M.G. The effect of ketamine/xylazine anesthesia on sensory and motor evoked potentials in the rat. *Spinal Cord* **41**, 16-22 (2003).

6. Hines, M.L. & Carnevale, N.T. The NEURON simulation environment. *Neural Comput* **9**, 1179-1209 (1997).

7. van Rossum, G. in Centrum voor Wiskunde en Informatica (CWI)1995).

8. Feinerman, O., Segal, M. & Moses, E. Signal propagation along unidimensional neuronal networks. *J Neurophysiol* **94**, 3406-3416 (2005).
